# Characterization of Rice Yield Based on Biomass and SPAD-Based Leaf Nitrogen for Large Genotype Plots

**DOI:** 10.3390/s23135917

**Published:** 2023-06-26

**Authors:** Andres F. Duque, Diego Patino, Julian D. Colorado, Eliel Petro, Maria C. Rebolledo, Ivan F. Mondragon, Natalia Espinosa, Nelson Amezquita, Oscar D. Puentes, Diego Mendez, Andres Jaramillo-Botero

**Affiliations:** 1 School of Engineering, Pontificia Universidad Javeriana Bogota, Cra. 7 No. 40-62, Bogota 110231, Colombia; 2 The OMICAS Alliance, Pontificia Universidad Javeriana, Cali 760031, Colombia; 3 The International Center for Tropical Agriculture CIAT, Km 17 Recta Cali–Palmira, Palmira 763537, Colombia; 4 Centre de Coopération Internationale en Recherche Agronomique pour le Développement (CIRAD), AGAP-Pam, Avenue Agropolis, 34398 Montpellier, France; 5 Fedearroz, Centro Experimental Las Lagunas, Km 4 Los Cairos, Tolima 730568, Colombia; 6 Chemistry and Chemical Engineering Division, California Institute of Technology, Pasadena, CA 91125, USA

**Keywords:** rice yield, UAV, multispectral imagery, vegetation indices, machine learning, nitrogen and biomass estimation

## Abstract

The use of Unmanned Aerial Vehicle (UAV) images for biomass and nitrogen estimation offers multiple opportunities for improving rice yields. UAV images provide detailed, high-resolution visual information about vegetation properties, enabling the identification of phenotypic characteristics for selecting the best varieties, improving yield predictions, and supporting ecosystem monitoring and conservation efforts. In this study, an analysis of biomass and nitrogen is conducted on 59 rice plots selected at random from a more extensive trial comprising 400 rice genotypes. A UAV acquires multispectral reflectance channels across a rice field of subplots containing different genotypes. Based on the ground-truth data, yields are characterized for the 59 plots and correlated with the Vegetation Indices (VIs) calculated from the photogrammetric mapping. The VIs are weighted by the segmentation of the plants from the soil and used as a feature matrix to estimate, via machine learning models, the biomass and nitrogen of the selected rice genotypes. The genotype IR 93346 presented the highest yield with a biomass gain of 10,252.78 kg/ha and an average daily biomass gain above 49.92 g/day. The VIs with the highest correlations with the ground-truth variables were NDVI and SAVI for wet biomass, GNDVI and NDVI for dry biomass, GNDVI and SAVI for height, and NDVI and ARVI for nitrogen. The machine learning model that performed best in estimating the variables of the 59 plots was the Gaussian Process Regression (GPR) model with a correlation factor of 0.98 for wet biomass, 0.99 for dry biomass, and 1 for nitrogen. The results presented demonstrate that it is possible to characterize the yields of rice plots containing different genotypes through ground-truth data and VIs.

## 1. Introduction

Rice is a staple crop in Colombia and plays a significant role in the country’s economy and food security. The average consumption of rice per capita in Colombia was 43.16 kg in 2021. According to the National Rice Growers Federation (FEDEARROZ), Colombia produced approximately 3.326 million metric tons of milled rice in the same year. The country’s rice production is concentrated in Tolima, Huila, Meta, Cauca, and Valle del Cauca. In Colombia, rice is primarily grown in two cropping seasons: a primary season from March to July and a second season from August to December [1]. The phenotypic expression of the rice varies depending on the interaction of the rice genotype with the environmental and growing conditions. FEDEARROZ defines five rice regions in the country with distinct environmental dynamics and high genetic variability in the weedy rice grown, resulting in highly diverse morphological characteristics [2].

Rice yields in Colombia vary depending on the location and production system, with higher yields typically achieved in irrigated systems. However, yields in rain-fed systems can also be high under favorable environmental and management conditions [3]. Various initiatives are in place to improve rice productivity and sustainability, including promoting high-yielding varieties and providing technical assistance and training to farmers [4,5,6]. One topic of interest is plant breeding, where, through the selection of favorable genes and higher-level omics characterization [7], optimal rice varieties are obtained for cultivation. To identify the important genetic characteristics expressed in the environment, phenotyping is performed in two directions: biomass accumulation and nitrogen content [8,9,10].

Biomass is a critical variable in rice crops, and estimating its accumulation in growing cycles enables crop-yield performance to be gauged, determining high-producing varieties [11]. The biomass estimation is usually made by cutting the plant, obtaining its fresh weight, and then dehydrating it to obtain its dry weight. An estimation of this variable using UAV imaging is a common approach that employs multispectral or hyperspectral sensors to capture information about the reflectance properties of vegetation in different parts of the spectrum [12]. This information can be used to calculate vegetation indices (VIs) from which biomass estimates can be derived. In [13,14,15,16], the effectiveness of this approach has been demonstrated, with biomass estimates obtained from UAV images showing strong correlations with on-the-ground measurements in different varieties of rice crops. In addition to providing accurate biomass estimates, UAV images can also be used to create detailed maps of biomass distribution, which can help identify spatial patterns and variability within crop fields [17].

Leaf-blade nitrogen concentration is correlated with the chlorophyll range in plants, and the variability of the pigments is another phenotypic characteristic. Nitrogen is an essential nutrient for plant growth and productivity, and accurate estimations of the nitrogen content in plants can lead to greater precision in fertilizer application, subsequently improving crop yields [18]. A correlation has been found between a plant’s leaf-blade nitrogen concentration and chlorophyll range. Given that the latter is a measurement of leaf-pigment variability, it can be estimated using UAV images. The detailed high-resolution visual data obtained from UAV images can be used to estimate the chlorophyll range and, therefore, indicate likely nitrogen distribution within rice crops. A common approach for estimating nitrogen using UAV images is to use spectral information to derive VIs related to nitrogen content [19]. These indices are based on the fact that radiation absorption in the visible and near-infrared spectra is related to nitrogen content in the vegetation. Several studies have demonstrated the potential of this approach, with UAV-based nitrogen content estimates showing strong correlations with on-the-ground measurements across various crops and ecosystems [9,20,21]. In addition to providing accurate estimates of plant nitrogen content, UAV images can also be used to create detailed maps of the nitrogen distribution within rice fields, which can help identify spatial patterns, variability, and guide precision fertilization [22,23].

Using UAV images for biomass and nitrogen estimation in rice crops is a promising approach for precision agriculture research [24]. By leveraging the high spatial and spectral resolution of UAV imagery, researchers can derive accurate and detailed estimates of these important variables, providing valuable information for understanding rice-crop dynamics [25]. Developing a model for biomass and nitrogen in rice is an important research topic within the area of food security, as rice is a staple crop that feeds more than half of the world’s population [26]. Accurately estimating biomass and nitrogen content in rice can help optimize crop management practices, increase yields, and reduce the environmental impact [27,28]. Developing non-invasive estimation methods, such as parameter estimation through multispectral imaging, allows for more frequent, automated estimates to be made, resulting in closer crop monitoring throughout the growing cycle and the possibility of more timely responses. A number of machine learning (ML) techniques have been employed to correlate VIs with biomass and nitrogen [29,30,31], including linear and nonlinear multivariate regression, support vector machine (SVM), and neural network (NN) models. However, there are other techniques that remain relatively unexplored such as decision trees, regression ensembles, and Gaussian regression processes.

This paper explores biomass and nitrogen dynamics in 59 rice plots and correlates these ground-truth measurements with information drawn from multispectral images captured at a height of 20 m. This study aims to (1) observe the dynamics of biomass and nitrogen behavior in the different genotypes sampled, establishing the ones with the highest yields; (2) analyze the behavior of the VIs in each plot in relation to its genotype; and (3) test various ML techniques to correlate the samples taken using traditional methods with the data drawn from the multispectral sensor images.

## 2. Materials and Methods

The experiment was conducted during the dry season in 2021 at Saldaña, which is a municipality located in the department of Tolima, Colombia. Situated in the Magdalena River Valley, Saldaña has fertile soils and a suitable climate for rice cultivation. Rice is one of the main agricultural crops in the region, and Saldaña is one of the top rice-producing municipalities in Tolima. Low-lying Tolima has a typically tropical climate, with average temperatures between 18 °C and 28 °C. It has two rainy seasons per year, one from March to May and the other from September to November, and two dry seasons, one from December to February and the other from June to August.

**Figure 1:**
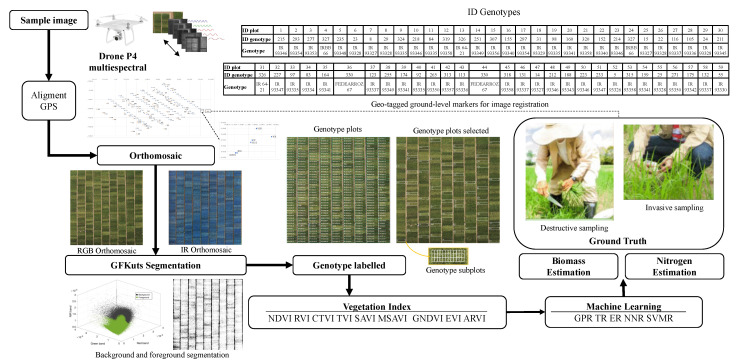
Methodology for canopy nitrogen and biomass estimation in large genotype rice plots using UAV multispectral images.

Samples were obtained from the FEDEARROZ (National Rice Growers Federation) experimental station Las Lagunas, in the plot located at latitude 3°54${}^{\prime }$55.29${}^{\prime \prime }$ north and longitude 74°59${}^{\prime }$02.75${}^{\prime \prime }$ west at 304 masl. The plot has 410 crop subplots, with an average crop area of 1.72 m${}^{2}$ each, and contains 330 different genotypes. At flowering, 56 genotypes were randomly selected based on their distribution in the experimental field. Of these genotypes, 53 had 1 repetition, whereas the other 3 had 2 repetitions due to their agronomic relevance. The sampling unit for the determination of biomass had an area of 0.2 m${}^{2}$ (5 plants per linear meter). Figure 1 shows the GPS points and the distributions of the genotypes in the experiment. The images taken by the UAV are aligned and orthorectified in two orthomosaic maps, the first in the visible light spectrum with red, blue, and green RGB channels, and the second with the red, red-edge, and near-infrared channels. The genotype of interest is labeled with its GPS points and the image extracted to relate it to the biomass and nitrogen measurements in the crop. The genotype image dataset consists of 2475 images in each channel. This set of images was used to evaluate five models for estimating biomass and nitrogen parameters by calculating VIs.

### 2.1. Experiment Sampling

Sampling was conducted using two methods: destructive or invasive methods and non-destructive or non-invasive methods. The first method consisted of harvesting the entire plant above ground level. The plants were weighed to determine their fresh weight and later the organs were separated into their different parts (such as stems, leaves, and panicles. The plants were dried in an oven at 65 °C for 72 h and weighed to determine the total dry weight of the plant. The values of both the fresh and dry weights were used to determine the percentage of water content (WC) of the plant for each genotype according to:

(1) $$\%WC=\frac{Weight_{fresh}-Weight_{dry}}{Weight_{fresh}}\times 100$$

This method is considered the most accurate but time-consuming. In addition, 5 plants per plot were selected due to the amount of plant material to be processed, and 59 samples were obtained to determine the biomass using this method. Plant height was also measured as an indicator of biomass [32,33].

Nitrogen levels were determined using a chlorophyll meter to measure the relative chlorophyll content in the leaves. These meters emit light of a specific wavelength onto the leaf, and the amount of light absorbed by the chlorophyll is measured. The meter reading can then estimate a plant’s nitrogen content [34]. The rice plots were sampled using a SPAD 502 Plus meter (Konica-Minolta), and the ground truth was established using this measure to correlate with the VI estimates.

The values obtained in the field through the first sampling method are displayed in Table 1 and Table 2. The variables include fresh weight, dry weight, water percentage, SPAD measurement, and height of the selected rice crops.

The second method involved instruments and techniques that did not destroy the plants, allowing for repeated measurements and, therefore, monitoring over time. The sampling was carried out by a UAV, which captured multispectral images to estimate the biomass and nitrogen content in the rice crops. The channels of each camera can detect differences in the reflectance of light at different wavelengths, which can be correlated with the biomass and nitrogen content in the plants [35].

**Figure 2:**
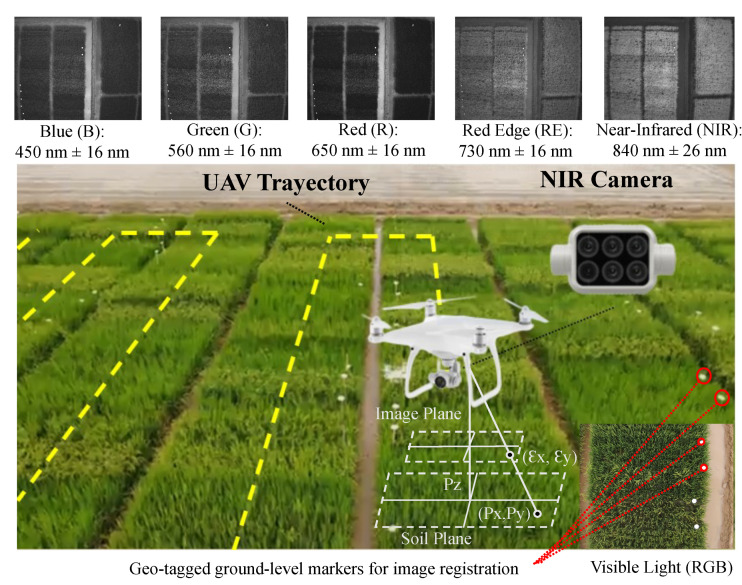
Non-invasive sampling method using multispectral images.

Figure 2 illustrates the tools employed for acquiring the multispectral images and the outcome of each channel. The UAV followed the yellow trajectory across each row of rice plots. The rice plots were georeferenced according to the flight altitude and geo-tagged ground-level markers. With each separate spectral sensor, the camera offered a resolution of 1600 × 1300 pixels, translating to a crop-to-image resolution of 2.5 cm/pixel at a flying height of 20 m.

The selected UAV was the Phantom 4 Multispectral, which had six cameras, each with 1/2.9-inch CMOS sensors, including an RGB camera and a multispectral camera array with five cameras covering the blue (B), green (G), red (R), red-edge (RE), and near-infrared (NIR) bands, with wavelengths of 450 nm, 560 nm, 650 nm, 730 nm, and 840 nm respectively. The uncertainty in each channel was ±16 nm. The precision of the multispectral data obtained was maximized by the spectral sunlight sensor on top of the aircraft, which could detect solar irradiance in real time for image compensation. To prevent aberrations that could occur when employing a rolling shutter, the P4 Multispectral used a global shutter.

The acquisition of the multispectral images was carried out on 14 September 2021, from 9:45 a.m. to 12:10 p.m., and the traditional sampling of the other parameters was conducted in parallel. The sampling was carried out on these dates because it was 90 days after sowing, which is the average period during which all genotypes are in the reproductive stage, allowing for the collection of samples at an intermediate state in plant growth. The drone sampling was conducted in a single flight, capturing 588 images with the 6 cameras (RGB, R, G, B, IR, NIR) at 98 points within the crop.

The images were orthorectified with the camera parameters derived from the camera and its position to correct any geometric distortions in the image. This process was aimed at removing any potential distortions and perspective effects from the images, as distortions can significantly impact the precision of the orthomosaic process.

The interior orientation parameters (IOPs) describe the technical specifications of the camera, such as the focal length, location of the principal point, and lens distortion coefficients. These parameters were used to correct distortions caused by the camera’s lens and sensor. Equations (2) and (3) illustrate the orthocorrected pixel coordinates [36].

(2) $$X=\frac{(x-c_{x})\cdot H}{h}-\frac{\left(y-c_{y}\right)\cdot tan\left(\phi \right)\cdot H}{h}+X_{0}$$

(3) $$Y=\frac{(y-c_{y})\cdot H}{h\cdot cos(\phi )}+Y_{0}$$

where *X* and *Y* are the orthocorrected pixel coordinates in the image, *x* and *y* are the original pixel coordinates in the image, $c_{x}$ and $c_{y}$ are the coordinates of the image center, *H* is the height of the camera above the ground, *h* is the image height in pixels, ϕ is the pitch angle of the camera (in radians), and $X_{0}$ and $Y_{0}$ are the coordinates of the image center in the ground coordinate system.

### 2.2. Feature Extraction

**Figure 3:**
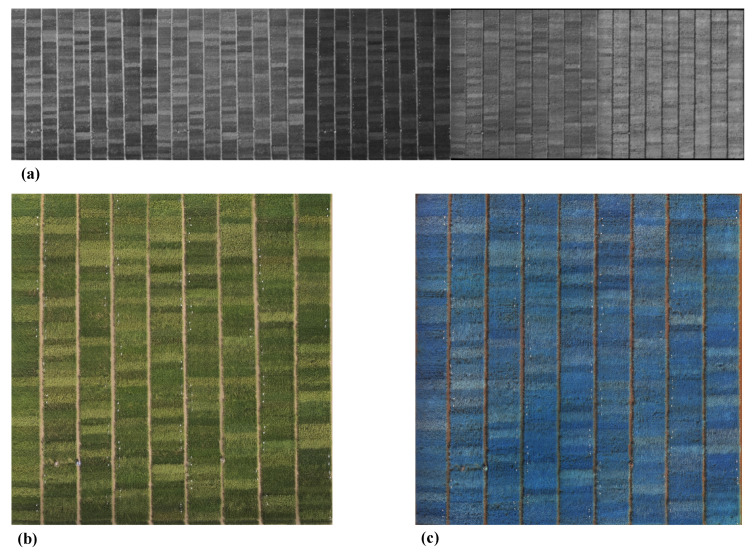
Preprocessing of the 98 images captured across the red, green, blue, red-edge, and near-infrared channels. (**a**) Orthomosaic of the different channels (red, green, blue, red-edge, and near-infrared, (**b**) Orthomosaic red-green-blue. (**c**) Orthomosaic red, red-edge, near-infrared.

In the feature extraction process, the characteristics of the selected rice crop were extracted from the images captured across all channels. An orthomosaic was then created from the 98 images captured from the five channels, as shown in Figure 3a. The orthomosaic was created using the geo-tagged ground-level markers depicted in Figure 2 to ensure that all channels were aligned when subsampling was performed in the plot. The channel data were combined and registered into two images for the purpose of visualizing the orthomosaic. The first orthomosaic, shown in Figure 3b, contains the red, green, and blue channels. The second orthomosaic, displayed in Figure 3c, includes the red, red-edge, and near-infrared channels. In the figures, each rice plot is subdivided into 40 subplots, each of which contains an average of one plant.

In order to ensure precision in the extraction of features, a segmentation method called GFkuts was applied, which has been used to accurately estimate biomass in other crops [15]. For the feature extraction from the UAV images, two methods were selected: the calculation of VIs and the use of the pixel averages of the multispectral images. A VI is a numerical value used to describe the health, density, or growth of vegetation in a particular area. Eight VIs were selected based on their relationship with biomass, nitrogen, and the spectral bands captured by the sensors used. The calculation of the VIs is presented in Table 3 and is explained in [35,37].

The GFKuts segmentation algorithm comprises multiple parts, including K-means clustering, GrabCut, and guided filtering. K-means is an unsupervised clustering algorithm that partitions an image into K clusters. It minimizes the within-cluster variance, which can be defined as follows:

(4) $$\sum_{i=1}^{k}\sum_{x\in S_{i}}||x-\mu_{i}{\left|\right|}^{2}$$

where x is a point in the image, $S_{i}$ is the set of points in cluster *i*, and $\mu_{i}$ is the mean of points in cluster *i*.

The GrabCut algorithm can be modeled as an energy-minimization problem. The energy of a labeling f can be defined as the sum of a region term R(f) and a boundary term B(f):

(5) E(f)=R(f)+λB(f)

where *f* is the label field and λ is a parameter that balances the two terms. The region term R(f) is the sum of the negative log-likelihoods of the color model for each pixel. The boundary term B(f) is defined in terms of the edges in the graph.

The output of the guided filter $q_{i}$ for an input image *I* and a guidance image *p* is defined as:

(6) $$q_{i}=a_{k}I_{i}+b_{k}$$

where for every pixel *i* in a box window *k*, $a_{k}$ and $b_{k}$ are linear coefficients that are the solution of the following minimization problem:

(7) $$\min_{a_{k},b_{k}}\sum_{i\in \omega_{k}}\left({\left(a_{k}I_{i}+b_{k}-p_{i}\right)}^{2}+\epsilon a_{k}^{2}\right)$$

where ϵ is a regularization parameter and $\omega_{k}$ is the window centered at pixel *k*.

In the GFKuts algorithm, K-means clustering is first applied to the image to generate an initial segmentation. This segmentation is then refined using GrabCut. Finally, guided filtering is used to smooth the segmentation result. The final output of the GFKuts algorithm is a binary mask that separates the rice canopy from the background.

Four feature matrices were proposed. The first two involved estimations using the genotype ID: the first feature set (FS1) used the aforementioned VIs, and the second feature set (FS2) used the mean pixel value of each multispectral channel and the segmentation. The last two involved estimations using the height: the third feature set (FS3) used the VIs and multispectral channels, and the fourth feature set (FS4) used the mean segmentation value. These feature matrices were designed to evaluate the usefulness of calculating the vegetative indices and the mean of the channels for estimating the variables. Once the four sets of features were obtained, they were labeled with the wet biomass, dry biomass, and SPAD estimation variables.

### 2.3. Estimation Models

The ground-truth data for training these ML algorithms are specified in Table 1 and Table 2. The data include the fresh weight, dry weight, percentage of water content, and measured SPAD values, which, through their adherence to a linear correlation, were directly correlated with the leaf-blade *N* concentrations. A number of plants that matched the previously mentioned plots were manually collected for destructive biomass testing. The samples taken from each subplot were weighed at the time of cutting for a fresh weight estimate, and again after drying to produce a dry-weight estimate in order to define the corresponding ground truth.

The collected multispectral image database consisted of 11,800 images, resulting from the 59 rice plots and the 40 subplots in each rice plot, totaling 2360 images in each channel. For the experiments, a cross-validation approach was proposed, using 80% of the data for cross-validation, amounting to a total of 1888 images. The remaining 20% of the data were used for testing and were not involved in the training process at all. From the 1888 images, five k-folds were randomly assembled, with each fold comprising 70% of the data for training and 30% for validation in order to enhance the robustness of the model. Training was performed for five models, including Gaussian process (GP) regression, tree regression (TR), ensemble regression (ER), support vector machine (SVM) regression, and neural network regression (NNR).

Tree regression is an ML technique in which a decision tree model is created to predict a continuous target variable. In this approach, the tree is constructed by categorizing the data into subsets based on the value of one of the input features. The goal is to recursively create binary splits that maximize the reduction in the variance of the target variable. The final model is a tree structure, where each leaf node contains a predicted value for the target variable [46]. Tree regression can be prone to overfitting so techniques such as pruning or ensembling multiple trees can be used to improve its performance.

(8) $$\mathrm{MSE}\left(t\right)=\frac{1}{N_{t}}\sum_{i\in D_{t}}{\left(y_{i}-{\bar{y}}_{t}\right)}^{2}$$

where MSE(t) is the mean square error at node t, $N_{t}$ is the total number of samples at node t, $D_{t}$ is the dataset at node t, $y_{i}$ is the target value of the i-th instance at node t, and ${\bar{y}}_{t}$ is the average value of the responses at node t.

Ensemble regression involves combining multiple models to make a more accurate prediction. One popular ensemble technique is random forest, which is an extension of the decision tree model. It creates multiple trees on randomly sampled subsets of the data and combines their predictions by averaging. Another popular ensemble technique is gradient boosting, which builds a sequence of decision trees in which each new tree attempts to correct the errors made by the previous trees [47]. Ensemble regression techniques can be more accurate than a single decision tree or linear model.

(9) $$\hat{y}\left(x\right)=\frac{1}{M}\sum_{m=1}^{M}f_{m}\left(x\right)$$

where $\hat{y}$(**x**) represents the ensemble prediction for an input x, *M* is the number of base regression models in the ensemble, and $f_{m}\left(x\right)$ is the prediction of the m-th base regression model for input x.

Gaussian process (GP) regression is a probabilistic machine learning technique used for regression problems. It is based on the assumption that any finite set of points in the input space has a joint Gaussian distribution over the corresponding target values. In GP regression, a prior distribution over the space of functions is defined using a covariance function, which determines how correlated the output values are for any two inputs. Given a set of input–output pairs, the posterior distribution over functions can be computed, which can be used to make predictions or calculate uncertainty estimates [48]. A Gaussian process is defined by a mean function m(x) and a covariance function (kernel) k(x, x′):

(10) $$f\left(x\right)∼\mathrm{GP}(m\left(x\right),k\left(x,x^{\prime }\right))$$

where f(**x**) represents the function value at input **x**, GP denotes a Gaussian process, m(x) is the mean function, (which is often assumed to be zero for simplicity), and k(x, x′) is the covariance function (kernel) that defines the relationship between different input points.

Support vector machines can also be used for regression problems. In SVM regression, the goal is to find a hyperplane that maximizes the margin between the predicted values and the actual values. The model tries to fit a linear function to the data while minimizing the errors or deviations from the target variable [49]. In cases where a linear function is inadequate for accurately fitting the data, a nonlinear kernel can be used to transform the data into a higher-dimensional space, where a linear function can better separate the data.

(11) $$\mathrm{minimize}\;\frac{1}{2}{\left||w|\right|}^{2}+C\sum_{i=1}^{N}\left(\xi_{i}+\xi_{i}^{*}\right)$$

(12) $$\mathrm{subject}\;\mathrm{to}\;y_{i}-w^{T}\phi \left(x_{i}\right)-b\leq \epsilon +\xi_{i}$$

(13) $$w^{T}\phi \left(x_{i}\right)+b-y_{i}\leq \epsilon +\xi_{i}^{*}$$

(14) $$\xi_{i},\xi_{i}^{*}\geq 0,\;i=1,\cdots ,N$$

where w is the weight vector, $\phi (x_{i})$ is a feature mapping function that maps the input vector $x_{i}$ to a higher-dimensional space, *b* is the bias term, *C* is a regularization parameter that controls the trade-off between maximizing the margin and minimizing the training error, $\xi_{i}$ and $\xi_{i}^{*}$ are slack variables that account for prediction errors outside the ε-tube, $y_{i}$ is the target value for the i-th instance, and *N* is the number of instances in the dataset.

Neural network regression models are a type of machine learning model that are well-suited for prediction tasks involving nonlinear data. They consist of an input layer, one or more hidden layers, and an output layer. Each layer consists of a number of nodes or “neurons”. Each neuron in a layer is connected to every neuron in the previous and subsequent layers [50]. The neurons transform the inputs using a weighted sum and a nonlinear activation function. The weights are learned during training by minimizing a loss function, such as the mean squared error for regression tasks, using an optimization algorithm such as stochastic gradient descent.

(15) $$y\left(x,w\right)=f\left(\sum_{j=1}^{M}w_{j}^{\left(2\right)}\sigma \left(\sum_{i=1}^{D}w_{ji}^{\left(1\right)}x_{i}+w_{j0}^{\left(1\right)}\right)+w_{0}^{\left(2\right)}\right)$$

where y(x,w) is the output of the neural network for the input vector x and weights w; *f* is the activation function of the output layer; σ is the activation function of the hidden layer; $w_{ji}^{\left(1\right)}$ and $w_{j}^{\left(2\right)}$ are the weights of the first and second layers, respectively; *D* is the number of input features; and *M* is the number of neurons in the hidden layer.

The performance of these machine learning models can be significantly influenced by the choice of hyperparameters. Hyperparameters are parameters whose values are set prior to the commencement of the learning process. To find the optimal hyperparameters, training is performed by varying these initial values to obtain the model with the minimum mean squared error (MSE). The hyperparameter optimization method selected for all the models was Bayesian optimization. This method can improve the search speed using past performances and achieve high accuracy with fewer samples [51]. The number of iterations for each model was defined based on the training time of each model. The models with the longest training times were Gaussian process regression (GPR) and neural network regression (NNR), with 5 and 300 iterations, respectively. Support vector machine regression (SVMR) followed, with 600 iterations. The models with the shortest training times were ensemble regression (ER) and tree regression (TR), with 800 and 900 iterations, respectively.

The evaluation of the estimations was conducted using regression metrics such as the coefficient of determination R², root mean square error (RMSE), and mean absolute error (MAE). The $R^{2}$ value was used to observe the fit between the estimated curves and the ground truth. The MAE provides a straightforward measure of the average magnitude of the error. One benefit of the MAE is that it is not overly sensitive to large errors, unlike the RMSE, which assigns a higher penalty to extreme values.

## 3. Results

The results were obtained from the selected and sampled genotypes, including the dry biomass, wet biomass, water percentage, height plant, and SPAD measurements. A total of 59 plots and 53 genotypes were sampled. Among these genotypes, the three genotypes IRBB, BR28, and Fedearroz had two samples each in different subplots, with varying sampling values. The remaining 50 genotypes had only one sample per plot. Sampling was carried out during two stages of the crop’s growth cycle: vegetative and harvest.

**Figure 4:**
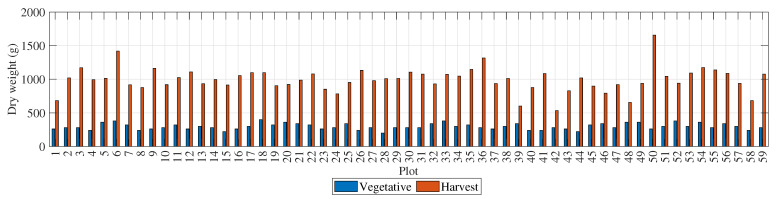
Total dry weight of each plot during the vegetative stage and at harvest time.

Figure 4 presents the dry-weight values for the 59 rice plots. The blue bars represent measurements taken during the vegetative stage, and the red bars represent measurements taken at the time of harvest. The dry weight is given in grams per plant, with an average of 296.27 g and a standard deviation of 45.70 g during the vegetative stage, and a mean of 994.20 g and a standard deviation of 181.03 g at the time of harvest. The plots with the highest dry weights during the vegetative stage were 18, 52, 33, and 6, with weights above 360 g. The plots with the highest dry weights at the time of harvest were 50, 6, 36, and 54, with weights above 1170 g.

The fresh-weight values shown in Table 1 and Table 2 are given in grams per plant, with a mean of 982.71 g and a standard deviation of 199.91 g during the vegetative stage, and a mean of 1244.44 g and a standard deviation of 2439.60 g at the time of harvest. The plots with the highest fresh weights during the vegetative stage were 34, 6, 5, and 54, all with weights for these genotypes of over 1300 g. The plots with the highest fresh weights at the time of harvest were 50, 6, 36, and 56, with weights of 1500 g. Both stages had similar total water weights but, as a proportion of the plant, the percentage of water was much higher during the vegetative stage. At the time of harvest when the crop was fully grown, the percentage of water was much lower with a higher proportion of biomass.

**Figure 5:**
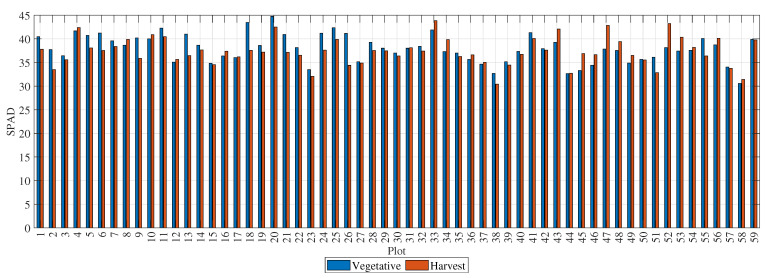
SPAD values of each plot at different times.

Figure 5 presents the SPAD values for the 59 rice plots. The blue bars represent measurements taken during the vegetative stage, and the red bars represent measurements taken at the time of harvest. The SPAD values had a mean of 37.97 and a standard deviation of 2.94 during the vegetative stage, and a mean of 37.36 and a standard deviation of 2.93 at the time of harvest. The plots with the highest SPAD values during the vegetative stage were 18, 20, 33, and 52, whereas the plots with the highest SPAD values at the time of harvest were 20, 18, 25, and 11. The SPAD value remained stable between the vegetative and harvest stages, indicating that the crop was adequately nourished throughout its growth period.

In order to determine the genotypes with the highest yield, both the yield per area and the biomass gain were calculated. The plots with the highest yields were 50, 36, 6, and 54 with 10,252.75 kg/ha, 8702.85 kg/ha, 8600.76 kg/ha, and 8330.06 kg/ha, respectively. For the biomass gain, the baseline was taken as the date of sampling during the vegetative stage and compared to the final biomass at the time of harvest. The greatest biomass gain was observed in plots 50, 6, 56, and 28, with averages of 49.91 g/day, 47.27 g/day, 46.78 g/day, and 40.44 g/day, respectively.

**Figure 6:**
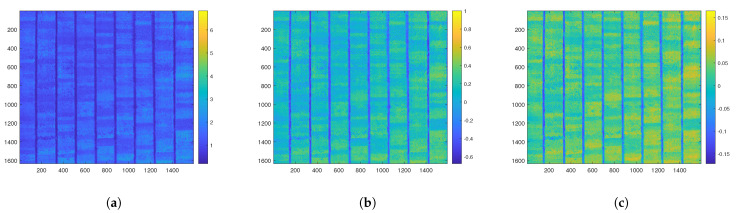
Distribution of the vegetative indices in the total rice crop. (**a**) Heatmap of the SR index. (**b**) Heatmap of the NDVI index. (**c**) Heatmap of the TVI index.

The orthomosaics were composed of 400 plots of different genotypes, and the first step in their processing was to locate them using the same spatial reference, allowing for alignment with the same reference point. Although not all 400 genotypes were sampled, an analysis of the VIs was performed on the entire orthomosaic. Figure 6 shows the VIs with the most significant differences in their values. The alignment and orthorectification allowed for the VI statuses to be mapped for the different genotypes. Although the entire crop was rice, significant variations in the VIs were observed. These variations were not necessarily due to different biomass and nitrogen values but rather to the phenotypic expressions of the cultivated genotypes. For rice crops of the same genotype, exploring the VI maps can be useful since they can reveal anomalies in the crops. The phenotypic expression should be similar and vary only due to changes in biomass or nitrogen.

**Figure 7:**
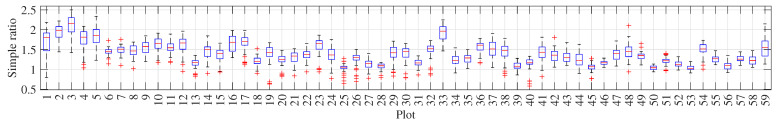
Box plot distribution for the SR vegetative index. Outliers are presented in the red values.

VI calculations were performed on the segmented image dataset of the 59 sampled plots. Figure 7 shows a box plot for the simple ratio (SR) index, which ranged from 0.5 and 2.5. This allowed us to observe the behavior of the simple ratio indices of the 59 selected genotypes and the uncertainty associated with each index. It was observed that genotype 1 had high variations, which further complicated its estimation. The plots with the highest values of this index were 3, 33, and 16. The plots with the most significant variations were 4, 24, and 26.

**Figure 8:**
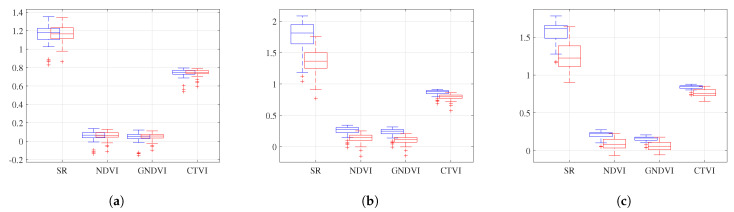
Box plots for the plot samples by genotype. (**a**) Box plot for genotype IRBB 66. (**b**) Box plot for genotype Fedearroz 67. (**c**) Box plot for genotype IR 64-21.

Figure 8 presents a box plot of the SR, DVI, GNDVI, and CTVI values for the three genotypes that had two plots each. These indices were selected for visualization because their range of variation was similar. The other indices exhibited smaller variations and similar behavior. These genotypes had a range of variation since the VIs were not uniform across the entire plot. However, sampling was only performed on one plant, assuming that the rest of the plants would have similar values, given that they belonged to the same genotype under the same growing conditions.

For the genotype IRBB 66, the SR average was 1.23 for plot 4 and 1.2 for plot 24, with a standard deviation of 0.24. The range was from 1.1 to 1.3, with outliers between 0.8 and 0.9 for plot 4. This plot had a fresh-weight value of 700 g, a dry-weight value of 240 g, and a SPAD value of 41.68. The range for plot 24 was from 1 to 1.2, with a fresh-weight value of 780 g, a dry-weight value of 280 g, and a SPAD value of 41.2. Although there was a directly proportional relationship with the SPAD values, the relationship with the biomass values was inversely proportional. It is essential to note that the variation between the VIs was low, as was the variation between the ground-truth values.

For the genotype Fedearroz 67, the SR average was 1.65 for plot 36 and 1.4 for plot 44, with a standard deviation of 1. A range of 1.2 to 2.1 was recorded, with outliers between 1 and 1.2 for plot 36. This plot had a fresh-weight value of 1000 g, a dry-weight value of 280 g, and a SPAD value of 35.66. The range for plot 44 was from 0.9 to 1.6, with a fresh-weight value of 660 g, a dry-weight value of 220 g, and a SPAD value of 32.64. This genotype had a directly proportional relationship between the SPAD values and the biomass values. In addition, the variation between the VIs was significant, as was the variation between the ground-truth values.

Finally, for the genotype IR 64-21, the SR average was 1.62 for plot 13 and 1.4 for plot 31, with a standard deviation of 0.6. The range was from 1.4 to 1.8, with the outliers at 1.1 for plot 13. This plot had a fresh-weight value of 940 g, a dry-weight value of 300 g, and a SPAD value of 41. The range for plot 31 was from 0.9 to 1.6, with a fresh-weight value of 900 g, a dry-weight value of 280 g, and a SPAD value of 38.02. This genotype had an inversely proportional relationship with the SPAD values and a directly proportional relationship with the biomass values. The variation between the VIs was significant, as was the variation between the ground-truth values.

**Figure 9:**
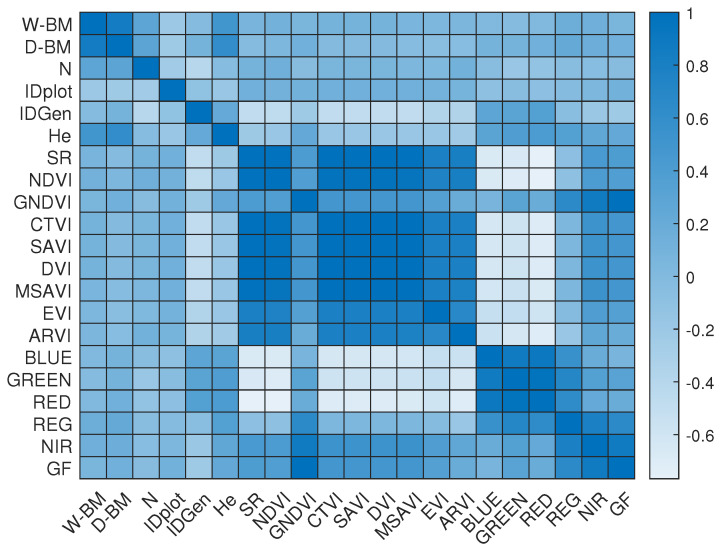
Correlation matrix of the selected features.

Figure 9 shows a correlation matrix for the dataset used, where W-BM is the wet biomass in grams, D-BM is the dry biomass, N is the SPAD value, and He is the average height of the plants. The VIs referenced in Table 3, the multispectral channels, and the GF segmentation values are also included. The correlation matrix revealed strong correlations among the data obtained using traditional methods. The VIs, which are mathematical operations between different reflectance values, also showed high correlations with each other. The VIs with the highest positive correlations with the ground-truth variables were the NDVI and SAVI for wet biomass, GNDVI and NDVI for dry biomass, GNDVI and SAVI for height, and NDVI and ARVI for nitrogen. The multispectral channels that correlated the most with wet biomass were REG, NIR, and GFkuts segmentation, whereas for dry biomass, the same channels were present but they also correlated with the RGB channels. Nitrogen showed fewer correlations with these channels and correlated more closely with the VIs. The ID genotype was highly correlated with the RGB channels, as well as the height. It can be observed that the height was highly correlated with the biomass variables, a valuable observation as it is an estimation parameter that does not require destructive methods.

As mentioned in Section 2, tests were conducted for five models. Each of the methods was optimized using Bayesian optimization, which can minimize a model’s confidence interval by adjusting the hyperparameters. The five models selected for training were evaluated using the four feature matrices. This process was executed using a common machine learning technique known as k-fold cross-validation, specifically with five randomly formed folds. In k-fold cross-validation, the original sample is randomly partitioned into k equal-sized subsamples. Of the k subsamples, a single subsample is retained as the validation data for testing the model, and the remaining k-1 subsamples are used as training data.

In this case, the folds were created from 80% of the total 2360 rice subplots, resulting in 1888 samples. These samples were used to train the models, taking into account the variability of the different datasets. These datasets were distributed in a 70/30 split, where 70% was used for training and 30% for validation. This split helps prevent overfitting, which is a modeling error that occurs when a function is too closely aligned to a limited set of data points.

In addition, 472 samples composed of 8 rice subplots of each genotype, were randomly set aside. These were not included in the model training and were reserved for the independent evaluation of the trained models. This is a necessary step to test how well the models generalize to unseen data.

In Table 4, Table 5 and Table 6, the values of the selected metrics, $R^{2}$, MAE, and RMSE, are presented. In general, it was found that decision tree regression, regression ensemble, and Gaussian process regression were the machine learning models that yielded the best correlations when trained on the data obtained during the experiment. Decision tree regression and regression ensemble are known for their efficiency and speed in training, whereas the Gaussian process regression, although it produces good results, is considerably slower.

The use of VIs improved the correlations for the estimations of the wet biomass, dry biomass, and nitrogen variables. The highest correlation among this set of training features was found when using the genotype ID, whereas the lowest correlation was associated with plant height. These correlations suggest that the genotype ID is a more significant predictor of crop yield.

**Figure 10:**
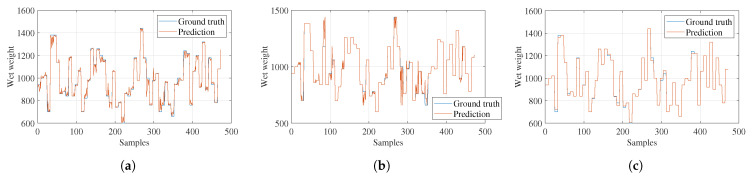
Models’ fresh-weight estimations. (**a**) Ensemble regression. (**b**) Tree regression. (**c**) Gaussian process regression.

The following figures show graphs of the estimations for the wet biomass, dry biomass, and nitrogen variables from the learning models with the highest correlation coefficients, with the estimated values in orange and the ground-truth values in blue. In Figure 10, the estimation for the first variable, wet biomass, is displayed. The results are shown for the training set of 472 images for the ensemble regression, tree regression, and Gaussian process regression estimation models. The graphs present the wet biomass of the samples (Y-axis) versus the samples obtained (X-axis) from each of the characterized images. In general, Gaussian process regression performed the best, obtaining a correlation coefficient of 0.987. This indicates that this model handles uncertainty much better for smaller datasets.

**Figure 11:**
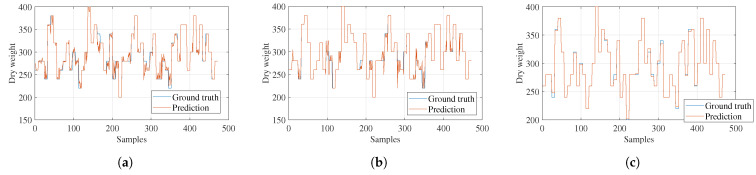
Models’ dry-weight estimations. (**a**) Ensemble regression. (**b**) Tree regression. (**c**) Gaussian process regression.

In Figure 11, the estimations for the second variable, dry biomass, are displayed. In general, better performance was observed for Gaussian process regression, which obtained a correlation coefficient of 0.998. This indicates that this model handles uncertainty much better for smaller datasets.

**Figure 12:**
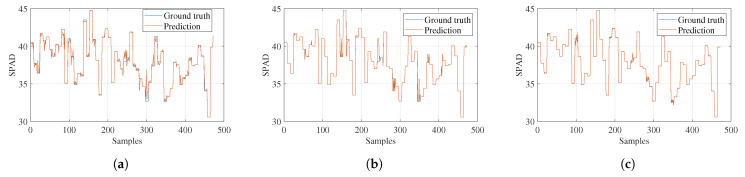
Models’ SPAD estimations. (**a**) Ensemble regression. (**b**) Tree regression. (**c**) Gaussian process regression.

In Figure 12, the estimations for the third variable, SPAD, are displayed. Better performance was observed for Gaussian process regression, which obtained a correlation coefficient of 0.999. This indicates that this model handles uncertainty much better for smaller datasets.

## 4. Discussion

The comparison of the GPR, SVMR, TR, ER, and NNR techniques provides valuable insights into the potential benefits and drawbacks of each method in the context of estimating the wet biomass, dry biomass, and nitrogen content in rice plots.

One notable aspect of this comparison is the emphasis on the probabilistic nature of GPR. In many practical applications, having a measure of uncertainty about the predictions can be just as important as the predictions themselves. Especially in agricultural applications, this measure of uncertainty can support decision-making tasks in the context of risk. For instance, knowing the uncertainty around the estimated nitrogen content in a rice plot can inform decisions about fertilization that balance potential yield improvements against the risk of over-fertilization and its environmental repercussions.

However, the computational cost of GPR, particularly for large datasets, is a significant drawback. As technological advances facilitate the collection of more and more data through increasingly sophisticated remote sensing technologies, the scalability of models becomes a critical concern. In this respect, the TR and ER methods may have an advantage.

The ability of ER and TR to handle complex, nonlinear relationships using decision rules is a significant advantage, considering the complex interactions among environmental factors, plant physiology, and the resulting variables to be estimated (biomass, nitrogen content). However, the careful selection of the kernel function and fine-tuning of hyperparameters can be potential drawbacks, as these require additional computational resources and domain expertise.

Ensemble techniques, which combine multiple base regression models, can potentially offer robustness and improved predictive performance, which is a significant advantage. Additionally, in this study, the estimation models were calibrated and tested by including both time-independent imagery samples and time-dependent vegetation index dynamics throughout each phenological cycle, enabling the characterization of spatio-temporal variations in above-ground biomass and leaf nitrogen.

However, these methods can also pose computational challenges, as multiple base models need to be trained and combined. Additionally, the interpretability of these models can be lost or reduced, which could be a disadvantage in scenarios where understanding the model’s decision process is crucial.

Selecting the most suitable method depends on factors such as the size of the dataset, the complexity of the relationship between the VIs and target variables, and the importance of uncertainty quantification in the predictions. It is also important to consider the computational resources available, the level of domain expertise for model tuning and interpretation, and the specific decision-making context in which the model’s predictions will be used.

It is worth noting that these methods are not mutually exclusive and could potentially be combined in a hybrid approach. For example, one could use a TR or an ER model to handle the main predictive task and a GPR model to provide uncertainty estimates.

Finally, the choice of the model should not only be guided by theoretical considerations but also validated through rigorous empirical testing. Cross-validation and performance metrics are indeed crucial tools for assessing and comparing the predictive performance of the models. Furthermore, whenever possible, models should be evaluated not only on their predictive performance but also on their practical impact when employed for decision making in the field.

## 5. Conclusions

The orthomosaic application is a useful tool for working with UAV images, as it allows for the integration of images captured by the UAVs in a flight plan, merging them into a single image. For this application to work, the images must have an area of coincidence greater than 40%. This tool enables a general mapping of the crop to be obtained in the form of VIs, classifying the vegetation on the ground by reflectance levels in order to estimate plant health and nutrient distribution patterns. In this study, the variation observed within each genotype reflects the heterogeneous nature of agricultural fields. This emphasizes the importance of using robust statistical methods in analyzing remote sensing data for biomass and nitrogen content estimation.

Multispectral images are useful for estimating wet biomass, dry biomass, and SPAD. Through the use of VIs, characteristics can be identified that closely correlate with the results from in-the-field sampling, offering researchers a non-invasive alternative for measuring this parameter and potentially eliminating the use of destructive sampling methods. The correlation between the VIs and the parameters measured in the field will vary according to the genotype and its phenological expression. In this study, the correlation matrix for the dataset revealed strong relationships between the data gathered conventionally in the field and several VIs. In addition, the VIs that correlated the most with the ground-truth variables were NDVI and SAVI for wet biomass, GNDVI and NDVI for dry biomass, GNDVI and SAVI for height, and NDVI and ARVI for nitrogen.

Moreover, the multispectral channels, specifically REG, NIR, and GFkuts segmentation, showed significant correlations with both wet and dry biomass, with an additional correlation observed with the RGB channels for dry biomass. Nitrogen content exhibited a weaker correlation with these channels, instead presenting a stronger correlation within the VIs. The ID genotype was highly correlated with the RGB channels and height, indicating a relationship between genetic identity, coloration, and growth. These correlations highlight the potential of remote sensing data in estimating key parameters such as biomass and nitrogen content in rice plots and emphasize the value of non-destructive parameters such as height for these estimation tasks.

## Figures and Tables

**Table 1:** Average values of measured parameters in the field for rice plots 1–30.

Parameter															Values															
Plot ID	1	2	3	4	5	6	7	8	9	10	11	12	13	14	15	16	17	18	19	20	21	22	23	24	25	26	27	28	29	30
Fresh weight (g)	940	1000	1020	700	1380	1380	1140	860	880	840	1180	840	940	1060	700	820	980	1260	1120	1260	1200	1160	840	780	1060	740	780	600	860	840
Dry Weight (g)	260	280	280	240	360	380	320	240	260	280	320	260	300	280	220	260	300	400	320	360	340	320	260	280	340	240	280	200	280	280
Water percentage	72.3	72.0	72.5	65.7	73.9	72.5	71.9	72.1	70.5	66.7	72.9	69.0	68.1	73.6	68.6	68.3	69.4	68.3	71.4	71.4	71.7	72.4	69.0	64.1	67.9	67.6	64.1	66.7	67.4	66.7
SPAD	40.48	37.72	36.42	41.68	40.74	41.24	39.58	38.66	40.2	40.02	42.26	35.06	41	38.64	34.86	36.4	36.04	43.48	38.62	44.76	40.94	38.14	33.5	41.2	42.36	41.14	35.14	39.28	38.02	37
Height	84	105.2	96.2	87	117.2	145.8	111	96.8	106	109.4	104.4	122	99.4	90.8	104.6	95.8	98.2	113.6	98.8	116	117.2	97.2	95.8	90	115.2	96.8	129.6	104.4	100.8	122.6

**Table 2:** Average values of measured parameters in the field for rice plots 31–59.

Parameter															Value														
Plot ID	31	32	33	34	35	36	37	38	39	40	41	42	43	44	45	46	47	48	49	50	51	52	53	54	55	56	57	58	59
Fresh weight (g)	900	1180	980	1440	1180	1000	760	980	1040	700	760	960	760	660	940	1000	980	1240	1220	760	1060	1200	920	1320	900	1180	940	780	1080
Dry Weight (g)	280	340	380	300	320	280	260	300	340	240	240	280	260	220	320	340	280	360	360	260	300	380	300	360	280	340	300	240	280
Water percentage	68.9	71.2	61.2	79.2	72.9	72.0	65.8	69.4	67.3	65.7	68.4	70.8	65.8	66.7	66.0	66.0	71.4	71.0	70.5	65.8	71.7	68.3	67.4	72.7	68.9	71.2	68.1	69.2	74.1
SPAD	38.02	38.44	41.9	37.32	37	35.66	34.64	32.7	35.16	37.36	41.32	37.94	39.28	32.64	33.3	34.42	37.84	37.52	34.9	35.64	36.1	38.14	37.42	37.58	40.08	38.72	34.04	30.58	39.88
Height	106.6	116.2	113.8	118.8	131.4	111.8	94.4	109.2	131.4	100	99.2	93.6	106	97.6	106.8	152.4	108	109.2	121.4	108	118	131	119.6	133.6	112	129.2	103.4	88	91

**Table 3:** Vegetation indices used in the experiment.

Vegetation Index	Formula	Application
Difference Vegetation Index (DVI)	NIR−RED	This index distinguishes between soil and vegetation but does not take into account the difference between the reflectance and radiance caused by atmospheric effects or shadows [38].
Normalized Difference Vegetation Index (NDVI)	$\frac{NIR-RED}{NIR+RED}$	NDVI is a widely used vegetation index that measures the difference between the near-infrared (NIR) and red light reflected by vegetation. Healthy vegetation typically reflects more NIR light and less red light so a high NDVI value indicates a high level of vegetation density and productivity [39].
Green Normalized Difference Vegetation Index (GNDVI)	$\frac{NIR-GREEN}{NIR+GREEN}$	GNDVI is primarily used to estimate vegetation biomass and monitor vegetation health [40].
Soil-Adjusted Vegetation Index (SAVI)	$\frac{NIR-RED}{NIR+RED+L}\cdot \left(1+L\right)$	SAVI is widely used to estimate vegetation biomass and monitor vegetation health, especially in areas with high soil background noise [41].
Modified Soil-Adjusted Vegetation Index (MSAVI)	$\frac{2*NIR}{2}+0.5-\frac{\sqrt{{\left(2*NIR+1\right)}^{2}-8*\left(NIR-RED\right)}}{2}$	MSAVI is widely used to estimate vegetation biomass and monitor vegetation health in a variety of environmental conditions [35].
Corrected Transformed Vegetation Index (CTVI)	$\frac{NDVI+0.5}{\left|NDVI+0.5\right|}\cdot \sqrt{\left|NDVI+0.5\right|}$	CTVI is primarily used to monitor vegetation health and stress [42].
Simple Ratio SR	$\frac{NIR}{RED}$	SR is widely used to estimate vegetation biomass and monitor vegetation health [43].
Transformed Vegetation Index (TVI)	$\sqrt{\left|NDVI+0.5\right|}$	TVI is used to monitor vegetation health and stress, and is also sensitive to changes in vegetation structure and composition [35].
Enhanced Vegetation Index (EVI)	$G*\frac{\left(NIR-RED\right)}{\left(NIR+C1*RED-C2*BLUE+L\right)}$	EVI is used to monitor rice growth and canopy biomass [44].
Atmospherically Resistant Vegetation Index (ARVI)	$ARVI=\frac{NIR-(BLUE-\gamma *(RED-BLUE))}{NIR+(BLUE-\gamma *(RED-BLUE))}$	ARVI is widely used to estimate vegetation biomass and monitor sensitive changes in vegetation with atmospheric correction [45].

**Table 4:** Evaluation metrics for the implemented models for dry biomass estimation.

Model	FS1			FS2			FS3			FS4		
	R2	MAE	RMSE	R2	MAE	RMSE	R2	MAE	RMSE	R2	MAE	RMSE
GPR	0.998	0.0305	0.119	0.97	0.0562	0.189	0.95	0.115	0.345	0.93	0.214	0.759
ER	0.96	21.509	41.668	0.96	21.606	41.391	0.83	29.769	81.012	0.93	39.303	54.028
TR	0.94	14.293	49.829	0.9	15.319	61.489	0.83	29.769	81.012	0.77	44.02	94.11
NNR	0.48	114.4	140.6	0.62	95.14	125	0.54	102.5	134	0.65	90.54	110
SVMR	0.42	125.2	158.6	0.58	102.65	118.6	0.53	109.4	140	0.6	97.86	122

**Table 5:** Evaluation metrics for the implemented models for wet biomass estimation.

Model	FS1			FS2			FS3			FS4		
	R2	MAE	RMSE	R2	MAE	RMSE	R2	MAE	RMSE	R2	MAE	RMSE
GPR	0.987	0.59112	2.4142	0.96	0.653	2.681	0.95	3.4624	7.456	0.92	3.589	9.1563
ER	0.88	11.295	15.481	0.85	13.146	18.158	0.85	19.64	23.231	0.7	20.114	25.099
TR	0.95	0.4432	5.7246	0.83	10.146	20.4562	0.9	9.5462	13.562	0.75	19.456	21.025
NNR	0.75	24.056	28.245	0.68	24.256	29.256	0.59	23.251	31.256	0.43	27.156	33.254
SVMR	0.64	21.055	30.025	0.42	28.745	35.254	0.43	26.152	34.521	0.31	30.376	39.272

**Table 6:** Evaluation metrics for the implemented models for nitrogen estimation.

Model	FS1			FS2			FS3			FS4		
	R2	MAE	RMSE	R2	MAE	RMSE	R2	MAE	RMSE	R2	MAE	RMSE
GPR	0.999	0.0305	0.119	0.998	0.0562	0.189	0.95	0.115	0.345	0.93	0.214	0.759
ER	0.97	0.194	0.482	0.98	0.197	0.454	0.91	0.452	0.956	0.92	0.241	0.901
TR	0.97	0.282	0.531	0.97	0.198	0.523	0.9	0.458	0.898	0.9	0.526	0.918
NNR	0.77	0.793	1.082	0.79	0.642	1.325	0.61	0.889	1.185	0.62	1.101	1.004
SVMR	0.65	0.861	1.154	0.68	0.785	1.552	0.54	1.001	1.201	0.58	1.165	1.198

## Data Availability

The data presented in this study are available on request from the corresponding author. The data are not publicly available due to patent in progress.
